# Infrared video tracking of *Anopheles gambiae* at insecticide-treated bed nets reveals rapid decisive impact after brief localised net contact

**DOI:** 10.1038/srep13392

**Published:** 2015-09-01

**Authors:** Josephine E.A. Parker, Natalia Angarita-Jaimes, Mayumi Abe, Catherine E. Towers, David Towers, Philip J. McCall

**Affiliations:** 1 Vector Biology Department, Liverpool School of Tropical Medicine, Pembroke Place, Liverpool L3 5QA, UK; 2 Optical Engineering Group, School of Engineering, University of Warwick, Coventry, CV4 7AL, UK

## Abstract

Long-lasting insecticidal bed nets (LLINs) protect humans from malaria transmission and are fundamental to malaria control worldwide, but little is known of how mosquitoes interact with nets. Elucidating LLIN mode of action is essential to maintain or improve efficacy, an urgent need as emerging insecticide resistance threatens their future. Tracking multiple free-flying *Anopheles gambiae* responding to human-occupied bed nets in a novel large-scale system, we characterised key behaviours and events. Four behavioural modes with different levels of net contact were defined: swooping, visiting, bouncing and resting. Approximately 75% of all activity occurred at the bed net roof where multiple brief contacts were focussed above the occupant’s torso. Total flight and net contact times were lower at LLINs than untreated nets but the essential character of the response was unaltered. LLINs did not repel mosquitoes but impacted rapidly: LLIN contact of less than 1 minute per mosquito during the first ten minutes reduced subsequent activity; after thirty minutes, activity at LLINs was negligible. Velocity measurements showed that mosquitoes detected nets, including unbaited untreated nets, prior to contact. This is the most complete characterisation of mosquito-LLIN interactions to date, and reveals many aspects of LLIN mode of action, important for developing the next generation of LLINs.

Many of the important mosquito vectors of malaria feed indoors at night, where and when most human malaria is transmitted in Africa^1^,^2^. Long-lasting insecticidal bed nets (LLINs) exploit this behaviour and are one of the most effective methods for reducing malaria transmission, fundamental to malaria control (amounting to $2.5 bn in 2013) and to ambitious plans for its elimination^3^,^4^. However, their future is seriously threatened by emerging resistance in vector populations to pyrethroids, the only insecticide class that can be used with LLINs^5^,^6^ and the need for novel LLIN designs that enable safe use of other insecticides or entirely new control devices or strategies is a global health priority^4^.

Delivering the ‘next generation’ of LLINs or similar tools will require a thorough understanding of how LLINs function, yet remarkably little is known of the mode of action or of precisely how mosquitoes behave at the LLIN interface. Recent studies using ‘sticky-nets’ reported that host-seeking female *Anopheles spp.* landed preferentially on the top surface of bed nets^7^,^8^ but that lethal capture method recorded only a single landing event and no other behaviours before or after. Although clustering at the net roof is likely to be a response to an attractant ‘plume’ rising from the human beneath, this too remains speculative because knowledge of mosquito flight behaviour prior to blood-feeding and of the identity and location of the key attractants that mediate the host-seeking response is limited^9^,^10^,^11^,^12^. Importantly, how insecticide treatments influence that response is unclear. Some studies reported that insecticide residues repelled mosquitoes prior to contact^13^,^14^, which would reduce or eliminate the chance of mosquitoes receiving an effective dose and potentially divert them to unprotected hosts^15^. Others found no evidence for such repellency^16^,^17^,^18^,^19^ indicating that LLINs attract and impact on mosquitoes by direct contact.

A further complication is the existence of what is termed ‘contact-irritancy’ or ‘excito-repellency’, whereby brief exposure to an insecticide can result in mosquitoes exhibiting avoidance behaviour, potentially before a lethal dose has been delivered^13^,^20^. Remarkably, some basic details are missing: *e.g.* the minimum duration of LLIN contact necessary to deliver an effective dosage is not known. Despite these phenomena being recognised for decades^20^,^21^,^22^, when and how they occur and their relative importance in selecting for insecticide resistance have never been fully elucidated.

Consequently, behavioural resistance to insecticides remains poorly understood and rarely reported in mosquitoes, though the risk of vector populations switching blood-feeding times, locations or host preferences in order to avoid LLINs is recognized and closely monitored today^23^,^24^,^25^. However, additional but less apparent or detectable behavioural changes also might exist, potentially conferring partial or complete insecticide resistance (*e.g.* changes in sensitivity to repellents, attractants, or modified flight or resting behaviours). In the absence of definitions or quantifications of the basic behavioural events likely to be affected^26^,^27^, these changes cannot be investigated, let alone monitored.

Ideally, characterisation of mosquito behaviour requires direct observation under conditions that are as ‘natural’ as possible. Informative studies to date have been limited to wind-tunnel or small-scale laboratory tests, potentially restricting mosquito flight. Frequently, tests use artificial or incomplete attractants such as human breath or limited body parts, carbon dioxide, single attractant chemicals or simple odour blends^9^,^18^,^28^, rather than an entire human host. Experimental huts^29^,^30^,^31^, electrocution grids^32^,^33^, taxis boxes^34^ and other methods overcome some of these obstacles but are unsuitable for detailed exploration of behavioural sequences.

Addressing many of the technical challenges that hindered progress to date, we have developed and constructed a novel system that enables tracking, recording and analysis of the flight paths of multiple individual mosquitoes over long periods in the dark at large volumes around the entire human host. In this first application of the system, we describe the flight and landing behaviour of *Anopheles gambiae* responding to human hosts within bed nets in a large experimental laboratory, and we show that human-baited LLINs operate by luring mosquitoes into multiple brief contacts with the net, almost entirely on the net roof, within minutes of commencement of host seeking, virtually eliminating all flight activity by 30 minutes.

## Results

### Classification of mosquito behavioural modes

**Figure 1:**
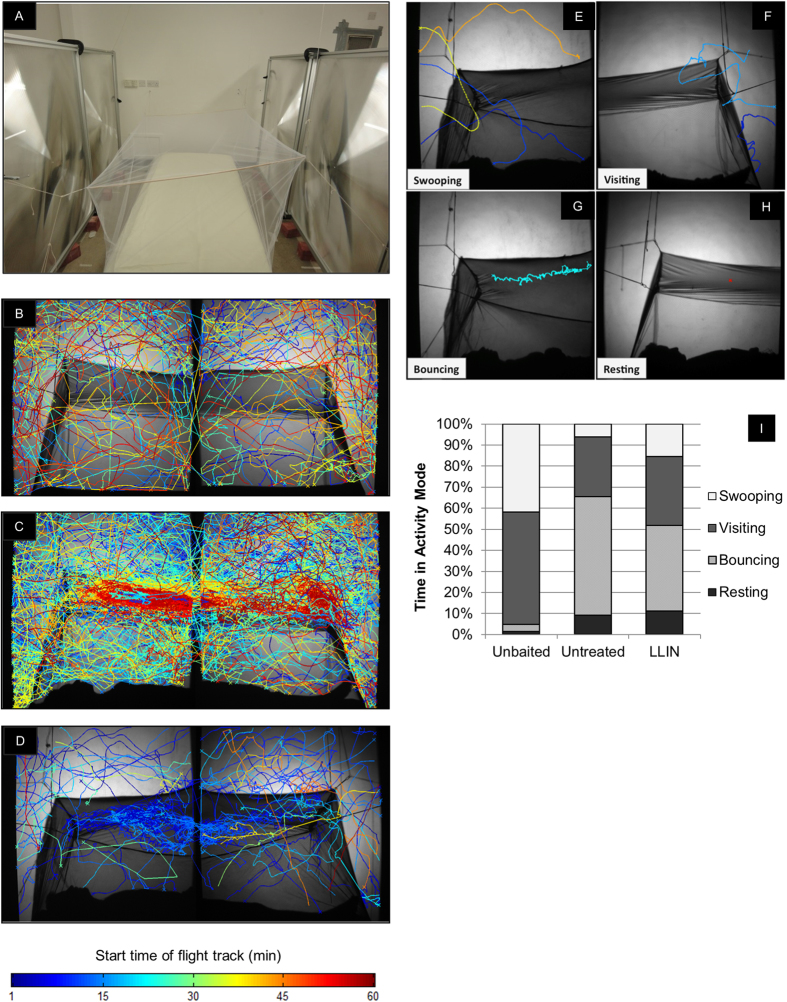
Flight activity of *Anopheles gambiae* at unbaited, baited and insecticide-treated bed nets. (**A**) The experimental insectary, showing the bed and fitted bed net, with two pairs of Fresnel lenses visible on the left and right; illumination at 850 nm was from two LEDs (not in image) located behind the right Fresnel lenses with the light from each forming an approximated parallel beam across the bed, through the left Fresnel lenses and focused into the camera beyond (not in photograph). The LEDs and camera were positioned 1.2 m behind the lenses, aligned to an optical axis through the centre of the Fresnel lenses. Mosquitoes were released on the wall behind the photographer at a height of 2 m. (**B**–**D**) Examples of a full 60-minute record of a test showing flight tracks of *Anopheles gambiae* at: (**B**) an unbaited untreated bed net; (**C**) a human-baited untreated bed net; (**D**) a human-baited insecticide-treated bednet (LLIN; Permanet 2^®^; Vestergaard-Frandsen, Lausanne, Switzerland). Twenty-five mosquitoes were released in all tests and activity was recorded for 60 minutes. Each coloured track is the path of a single mosquito flight event. Tracks are colour-coded according to time they first appeared in the field of view as shown in the key: blue tracks at the start through to red at the end of the 60-minute test. (**E–H**) Images showing representative tracks for *Anopheles gambiae* flight in each of the four behaviour modes as defined in the text. See also Supplementary Video. (**I**) The proportion of time spent in each behaviour mode for each bed net type: Unbaited = unbaited untreated bed net; Untreated = human-baited untreated bed net; LLIN = human-baited insecticide-treated bed net (LLIN).

In all treatments, mosquito activity was classified into four quantifiably distinct types of behaviours, termed ‘modes’ (Fig. 1E–H; Supplementary Video), defined as follows:

1. Swooping: Tracks that did not contact the bed net.
2. Visiting: Tracks where relatively long periods of flight were interspersed with infrequent contacts with the bed net. Contacts were characterized by sharp turns of 80° or more in the trajectory and when multiple contacts occurred, the minimum interval between them was 0.4 seconds (*i.e.* an interval of at least 20 frames, at 50 frames per second).
3. Bouncing: Tracks where the mosquito made multiple rapid contacts with the bed net surface, at intervals of less than 0.4 seconds; includes events where the mosquito executed short flights between contacts, or maintained contact with the bed net surface without being static. The latter were brief pauses in movement lasting less than 0.75 seconds and included ‘walking’ on and ‘probing’ the bed net.
4. Resting: Mosquito tracks where insects were either completely static for at least 0.75 seconds, or where the velocity of mosquito movement was less than 1.33 mm/s (equivalent to movement of up to one mm in the minimum resting time); assumed constant contact with the bed net surface. Dead mosquitoes were excluded by limiting resting events to a maximum of 300 seconds. Notably, no dead mosquitoes were found on nets at the end of tests.

### Responses at unbaited, baited and insecticide-treated nets

Figure 1B–D show representative examples of recorded flight tracks at unbaited untreated (henceforth termed ‘unbaited’), baited untreated (‘untreated’) and baited LLIN (‘LLIN’) nets during 1 hour of recording. Across all treatments, individual flight track durations ranged from 0.22 to 445.1 seconds, with a geometric mean track length of 4.2 seconds (4.0–4.3; n = 7729 tracks).

Activity at an untreated bed net was significantly lower in the absence of human bait, as measured by the mean number of flight tracks (212 [−78–501] at unbaited and 545 [410–679] at untreated nets; generalized linear model, *p* < 0.001) and the total duration of activity recorded for each test (*i.e.* 25 mosquitoes for 1 hour, maximum of 25 hours: geometric means of 19.0 [1.6–223.5] and 124.6 mins [101.5–153.0] at unbaited and untreated respectively; *p* < 0.001). Activity at LLINs (164.7 tracks [91–238]; 21.2 mins [10.6–42.7]) was significantly lower than at untreated nets (*p* < 0.001) but similar to unbaited nets (*p* = 0.456, *p* *=* 0.649 for track number and duration, respectively).

Exploring this activity by behavioural mode (Fig. 1I) shows that 93.7% of activity on a baited (untreated) net involved net contact (*i.e.* visiting, bouncing or resting modes) compared to 58.1% on an unbaited net. In fact, 65.3% of the activity at a baited net involved frequent (bouncing) or continuous (resting) net contact, in contrast to 4.7% on the unbaited net. Moreover, the mean times spent in each mode involving contact (Table 1) were significantly higher (visiting, *p* = 0.009; bouncing, *p* < 0.001; resting, *p* < 0.001) in the presence of human bait while swooping was not significantly different (*p* = 0.953). At LLINs, activity in all four behaviour modes was significantly lower than at untreated nets, particularly in the three modes with net contact where treated net activity fell to 27% or less than the untreated net values (Table 1, *p* < 0.001 except swooping, *p* = 0.002). However, a response to the host persisted despite the insecticide presence as evidenced by the values for bouncing and resting modes, which were significantly higher than at unbaited nets (*p* = 0.001, and *p* = 0.010).

### Flight speed, tortuosity and height during net approach

The instantaneous velocity of individual swooping flight tracks ranged from 83.9 to 985.8 mm/s across all tests, with a mean velocity of 346.2 mm/s (341.6–350.8; n = 3234 tracks). Mosquitoes flew slightly faster at baited untreated nets (355.8 mm/s [340.1–371.5]) than at unbaited nets (321.1 mm/s [265.8–376.4]; *p* = 0.005) and LLINs (322.7 mm/s [292.7–352.8]; *p* = 0.020), which were not significantly different from each other (*p* = 0.923).

Track tortuosity was higher in both baited net groups than in the unbaited nets (1.31 [1.16–1.47] unbaited, 1.66 [1.52–1.79] untreated, 1.63 [1.43–1.83] LLIN; *p* < 0.001), but not different between LLINs and baited untreated nets (*p* *=* 0.783).

Simple analysis of the spatial location of flight path prior to arrival did not indicate any notable bias for low (below top net surface level) or high (above the net) spatial preferences. Adjusting for differences in the visible field of view between high and low areas, equal distribution would result in 36% of tracks starting in the high region. In unbaited and untreated tests, there was no preference (38.0% [34.7–41.2] and 40.3% [32.8–47.9] of tracks starting above the net; *p* = 0.134, *p* = 0.237; one sample t-test), though in baited LLINs marginally more net approaches started above the net than below (41.5% [37.0–46.1]; *p* = 0.024).

### Location of activity at the bed net interface

**Figure 2:**
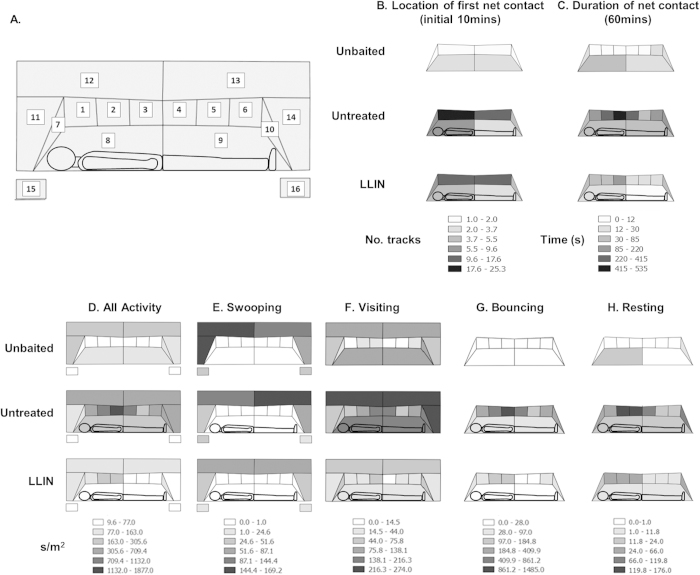
Distribution of *Anopheles gambiae* flight activity, behaviour modes and net contact at different regions on and around a bed net (**A**) Distribution map key showing region codes to which each mosquito track was assigned. Bed net surfaces 1–6 were on the horizontal roof, 7 and 10 the vertical head and foot end walls, respectively, 8 and 9 the vertical side walls. Portions of flight tracks visible beyond the net surfaces were assigned to the spatial regions 11–14 as shown. Regions 15 and 16 record flight activity without net contact (*i.e.* swooping) that occurred in front of net, on the left (15) and right (16) portions of the field of view, respectively. (**B**) Distribution of initial net contacts by region, showing the first point of net contact for those tracks occurring in the first ten minutes of testing. (**C**) The total duration (seconds) of all contacts (includes mid-flight brief contacts made during visiting and bouncing, and resting behaviour) by all mosquitoes on each region of the bed net surface over the 60 minute test (means of 3,10 and 10 replicate tests for unbaited, baited and LLIN, respectively). Charts D–H show the density of activity (s/m^2^) around and on the bed net surface: (**D**) All activity combined; (**E**) Swooping; (**F**) Visiting; (**G**) Bouncing; (**H**) Resting. Although tests controlled for the orientation of the human bait in relation to the mosquito release point, all figures show the volunteer (when present) with the head on the left.

The distribution of total activity (seconds/ m^2^) around the bed net was significantly different at each net type (*p* *<* 0.001)(Figs 2D and 1B–D). Without human bait, 49.9% of flights occurred in the spatial regions around the net (regions 11–16 in Fig. 2A), compared with 5.5% at untreated nets and 10.5% at LLINs (Fig. 2D). In contrast, activity in baited tests was located primarily on the net roof directly above the human body and to a lesser extent, near the feet: 74.7% and 78.3% of activity occurred on the roof (regions 1–6) and 10.9% and 8.8% at the feet (region 10) in untreated nets and LLINs respectively (Fig. 2D).

Comparing nets by behaviour mode, swooping (Fig. 2E) was distributed unevenly between different net regions in all treatments (*p* < 0.001), with less activity occurring in regions 15 and 16 in front of the vertical net sides. In visiting mode, there was a significant interaction between net type and activity distribution (*p* < 0.001; Fig. 2F): higher visiting rates were recorded in regions 3 and 4 (17% and 16% of activity) on untreated nets, but at LLINs, visiting was higher in regions 3, 7 and 10 (12%, 11% and 10%, respectively). Treatment also affected activity distribution in bouncing mode (*p* < 0.001): bouncing flight was higher in regions 2, 3 and 4 (21%, 35% and 17% respectively) in untreated nets, whereas most bouncing activity at LLINs occurred in regions 1, 2, and 3 (18%, 30%, 24%; Fig. 2G). Finally, net type also significantly affected resting activity (*p* < 0.001; Fig. 2H): on untreated nets, more resting occurred in regions 2, 3 and 10 (26%, 26%, 17%) but on LLINs, resting was higher in regions 1, 2 and 3 (17%, 20%, 33%), with 11% of resting recorded at the feet (region 10).

Hence, although there were marked significant differences between baited and unbaited nets for bouncing (*p* = 0.001) and resting (*p* = 0.001) modes (Fig. 2G,H), there was no evidence that insecticide treatment significantly altered the preference for the roof of the bed net as the focus of activity.

### Velocity of mosquitoes during landing on bed nets

The mean velocities of mosquitoes during final approach to the net surface, immediately prior to net contact, were determined for each test and compared between bed net types. In total, 896 tracks fitted the conditions for contact analysis. Of this subset, the mean percentages of net contacts classed as ‘contacts without deceleration’ (*i.e.* tracks that accelerated on their last two points of flight before contact or where deceleration did not start until within 3 mm of the net, and leg contact could not be excluded) were calculated as 1.7% [−2.0–5.3] at unbaited nets, 7.5% [5.6–9.4] at untreated nets and 7.6% [−0.1–15.3] at LLINs, and were not significantly different between treatments (*p* = 0.392). Hence, over 90% of mosquitoes decelerated prior to net contact, with deceleration starting at approximately 0.12 seconds prior to landing, at a distance of 26–41 mm from the net.

**Figure 3:**
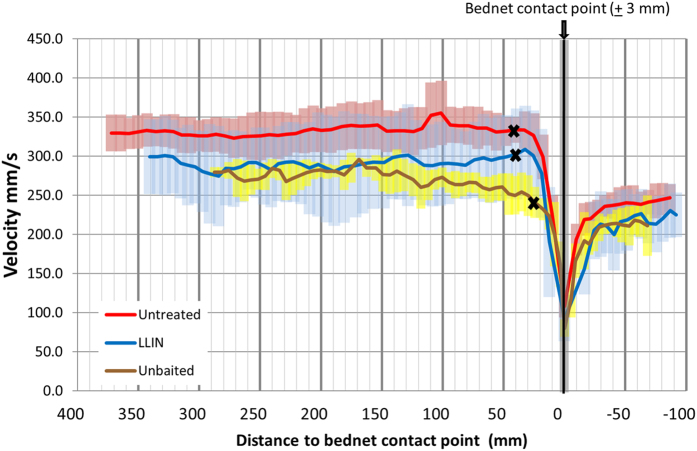
Velocity of *Anopheles gambiae* during landing at bednets. Mean velocity of mosquitoes during final approach, contact and departure from the bed net surface. The figure represents a 1.3 s track segment, with the bed net contact point at 0 mm; positive x-axis values indicate position before contact, negative values after contact. The grey region either side of the contact point represents the ± 3 mm region where tarsal contact with the bed net was possible. The average points at which deceleration started for each net type are marked with ‘X’. Note that the graph presents the averages of multiple repeat test values and hence the position of the point of deceleration does not correspond perfectly with the average approach track as illustrated. Coloured bars show standard deviation at each track point.

However, the point at which mosquitoes started to decelerate (Fig. 3) was significantly closer to the net at unbaited nets (distance from the net 26.3 mm [18.5–34.1]) than at baited nets, both untreated (41.5 mm [36.8–46.2], *p* = 0.010) and LLINs (40.0 mm [31.0–49.0], *p* = 0.019), which were not significantly different from each other (*p* = 0.708). In addition, unbaited arrival flight velocities (276.6 mm/s [212.6–340.7]) were significantly slower than those at untreated (384.4 mm/s [365.3–403.5], *p* < 0.001) and LLINs (356.9 mm/s [309.5–340.7], *p* < 0.007), which were not significantly different from each other (*p* = 0.175).

### Quantifying duration of net contact

The mean total time per test where mosquitoes were in physical contact with nets (Table 2) was significantly higher on the untreated baited net (33.1 min [24.3–42.0]) than on both the LLIN (7.3 min [3.9–10.7]; *p* *<* 0.001) and unbaited nets (2.4 min [−2.1–6.8]; *p* *<* 0.001; generalized linear model). However, contact time was significantly higher also on LLINs than on unbaited nets (*p* = 0.003). The longest contact time recorded for a single mosquito track was 37.4 s on an unbaited net, 160.4 s on an untreated net and 110.5 s on an LLIN. Since it was not possible to measure the actual total contact time for individual mosquitoes, we determined plausible mean minimum and maximum contact time values (as defined in Table 2) for a single mosquito of 79.5 to 334.1 seconds at an untreated baited net and 17.5 and 95.6 seconds at an LLIN over 60 minutes of a test.

Total contact time was significantly affected by interactions of net type and region (*p* < 0.001, *p* *<* 0.001; Fig. 2C). Highest contact times (extracted contact data, of all types, from all tracks) were recorded on the roof in untreated nets (regions 2, 3, 4: 410s, 531s, 306s, respectively), and in the centre of the roof in LLINs (region 3: 126s).

### Interactions with the bed net over time

**Figure 4:**
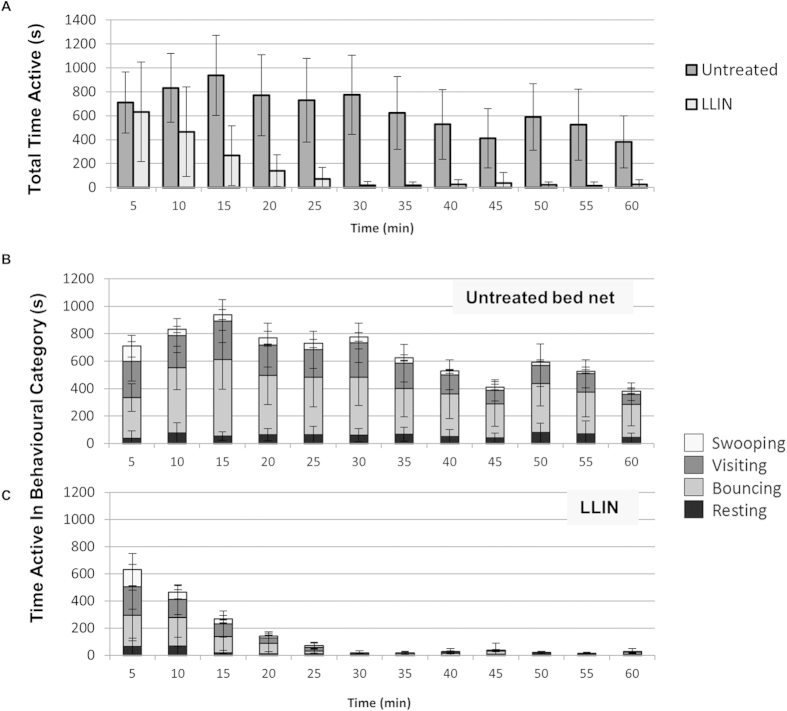
Rates of *Anopheles gambiae* activity throughout the 60 minute test period. (**A**) Total activity at untreated baited nets and LLINs.(**B,C**) Mosquito activity as in Fig. 3A separated by behavioural mode, at untreated baited nets (**B**) and LLINs (**C**). X-axis units are mean (±SD) activity per 5-minute inclusive interval, *i.e.* 5 (0–4 min 59 s), 10 (5 min–9 min 59 s), 15 (10–14 min 59 s), etc.

Over 60 minutes, total mosquito activity decayed significantly more rapidly at LLINs compared to untreated nets (*p* = 0.004, Generalized Linear Model; Fig. 4A). Reduced activity at the LLIN was indicated after 5–10 minutes, becoming significantly lower from the 10–15 minute period onwards. By 30 minutes, activity at LLINs was negligible and did not recover, while sustained levels of host seeking were recorded at untreated nets for the entire 60 minutes. This rapid fall was apparent in the three behavioural modes involving flight, swooping, visiting, and bouncing (*p* = 0.016, *p* = 0.031, *p* = 0.002, respectively, Generalized Linear Model; Fig. 4B,C).

Mosquito activity during this key initial 10-minute period was explored further. The time lag between appearance of the first mosquito and first net contact was unaffected by the presence of the insecticide or human bait: geometric means unbaited = 18s (0–994); baited = 6s (1–33)(*p* = 0.438); LLINs = 17s (6–43)(*p* = 0.432). Comparing untreated baited nets with LLINs, there were no differences in the number (72.8 [53.1–92.5] at untreated nets, 50.3 [28.6–72.0] at LLINs; *p* = 0.084) or the distribution of contacts on different net regions (*p* = 0.145), with a significant majority (*p* < 0.001) of first contacts on the net roof in both (60.6% and 56.5% in untreated and LLINs, respectively)(Fig. 2B). At unbaited nets in contrast, significantly fewer contacts (12.3 [2.3–22.4]; *p* < 0.001) occurred in a significantly different pattern (*i.e.* near uniform distribution) on the net (*p* = 0.018)(Fig. 2B).

These results indicate that LLINs did not repel mosquitoes to any significant level prior to net contact. Yet, while contact with LLINs was significantly lower than untreated nets over 60 minutes (Fig. 1I, Table 1), the majority of LLIN contacts occurred during the first ten minutes: 62.2% on LLINs (4.6 min [2.2–6.9]), 17.9% on untreated nets (5.9 min [4.0–7.8]). Moreover, this impact was preceded by surprisingly brief time in contact with the LLIN. We calculated that during the initial ten minute period, one mosquito made between 14.3 and 70.3 seconds of contact with an untreated net or 11.0 and 57.1 seconds with an LLIN (minima and maxima calculated as described in the previous section).

## Discussion

These results provide detailed insight into the behaviour of *An. gambiae* at an LLIN. On detection of a human host within a bed net (with or without insecticide), mosquitoes responded immediately in four distinct behaviour modes, with persistent attempts to reach the host resulting in multiple brief net contacts focussed on the net roof above the human torso. Behaviour at an LLIN retained the essential character of the response to untreated nets for the first ten minutes, during which time less than one minute of total contact was made with the LLIN. A rapid decay in all modes of activity resulted and after thirty minutes, mosquito activity was negligible and did not recover. Lag times to response and velocities and deceleration rates prior to net contact were similar in LLINs and untreated nets, demonstrating the absence of LLIN repellency. The results demonstrate that an LLIN is a highly efficient fast-acting baited insecticide trap.

These results were obtained with an optical imaging and flight-tracking system allows remote tracking, recording and quantitative analysis of multiple mosquitoes simultaneously flying without restriction in large fields of view over long periods while they respond to a complete human host in complete darkness. For studies at this scale, the system offers a number of advantages over other approaches. Despite their undoubted value, existing tracking systems, including some three-dimensional (3D) systems, are restricted (in relation to this study’s goals) in terms of temporal resolution and test arena size constraints, short recording durations (up to 15 minutes), the low numbers of mosquitoes that can be observed simultaneously (1–4 mosquitoes per experiment) or the need to use isolated host cues such as heat or odour rather than complete human baits^9^,^28^,^35^,^36^. Studies that track multiple mosquitoes have been restricted by short recording periods of less than 3 minutes or the ability to track only initial and final behavioural events^35^,^36^,^37^,^38^. An effective stereo video system tracked up to 25 mosquitoes in wild mating swarms^37^,^38^,^39^ but required sunlight to generate the images.

The findings are novel and a significant contribution to our understanding of mosquito behaviour generally, and specifically how it is targeted by LLINs. Although the LLIN tested is only one of many types commercially available today, the Permanet^®^ 2.0 is one of the most purchased and widely used LLINs in Sub-Saharan Africa^40^. Clearly further studies must investigate other LLINs.

The immediate and rapid effect of the LLIN and the low level of net contact required to achieve that has never been reported. Less than one minute of contact within the first 10 minutes reduced subsequent foraging such that all flight and host location activity was virtually eliminated by 30 minutes. Whilst acknowledging that a limited number of net contacts may not have been captured by the tracking system (*e.g.* potentially obscured by the host, net seams or wrinkles, or during processing) we consider this to be an accurate measurement of LLIN contact duration.

Activity at baited nets, both untreated and LLINs, was higher than at unbaited nets, particularly in the bouncing and resting behaviour modes when the highest levels of net contact occur. While activity over 60 minutes was lower at LLINs than at untreated nets, there was no difference in the number, distribution or duration of net contacts in the first 10 minutes. Furthermore, velocities measured immediately prior to net contact were virtually identical in both untreated nets and LLINs, with no indication that mosquitoes were repelled or deterred by the insecticide at close range. Finally, there were no significant differences in the time lag prior to the initial mosquito’s response, confirming a previous report^18^, and indicating there was no distant or spatial insecticidal effect on behaviour.

This finding partially allays fears that LLINs might divert unfed but still hungry mosquitoes to non-users of nets without any LLIN contact^41^,^42^,^43^,^44^. However, it remains to be determined whether the observed elimination of activity at the LLIN (Fig. 4) resulted from insecticide-induced knockdown or death, an irreversible sub-lethal flight or sensory impairment, or some other reversible condition. Contact irritancy and impairment of host seeking responses by deltamethrin have been described^45^,^46^ but we were unable to recapture sufficient mosquitoes to determine mortality rates or sub-lethal effects post-exposure. We calculated that an individual mosquito made on average between 17.5–95.6 seconds of contact with the LLIN (Table 2; although the true value depended on the proportion of released mosquitoes responding). Earlier tests with *An. gambiae* and deltamethrin-treated nets reported knockdown and death of some individuals following net contact times of only 0.4 seconds while others survived after 40 seconds of contact^18^. These results suggest that, in reality, the effects of LLINs on individual mosquitoes may be wide ranging in severity. Hence, although our results demonstrate clearly that host seeking ceases rapidly when an LLIN is used, determining the proportion of mosquitoes that survive and remain capable of locating and feeding successfully on a different host following contact with an LLIN, is an important next step.

An important additional point is that these (Table 2 and Spitzen *et al.*^18^) LLIN contact values derive from observed behaviour and are considerably lower than the WHO standard method used for LLIN evaluation^47^, where mosquitoes are forced into contact with treated surfaces for 3 minutes. Although further accurate data are essential to confirm this, the duration of exposure used in standard evaluation of LLINs may need to be re-examined to avoid any possibility of overestimating the effectiveness of any material being tested.

Our results emphasise the importance of the bed net roof^7^,^8^ by showing that it is the predominant first point of contact (Fig. 2B), the most commonly visited surface (Fig. 2C), and that most flight activity is also focussed at or around the roof, regardless of the flight path of the arriving mosquito (Fig. 2D–F and 1C,D,G; Supplementary video). Though there was some additional activity near the feet, activity at the net sides was very low (Fig. 2C,G) indicating that mosquitoes oriented primarily to putative olfactory and thermal attractants rising from the prone host^12^,^48^,^49^. Within hypothesised models of vector host location^12^,^28^,^48^,^49^, the mosquitoes tracked in this study were relatively close to the host throughout, and therefore likely to have been flying in response to ‘broad plumes’ of host cues that would ultimately lure them to the net. Without knowing the actual location of those ‘plumes’ or their boundaries, it is not possible at this stage to interpret the observed flight trajectories or assign them to recognised behaviours such as ‘casting’^28^, where mosquitoes exhibit counterturning on leaving the plume in order to relocate it, or where increased tortuosity and decreased velocity occur as mosquitoes attempt to locate the source of the attractant^50^,^51^,^52^.

The four newly described behaviour modes provide a means to measure and compare the effectiveness of different treatments, including repellents or attractants. We hope also that the results will contribute towards the identification of possible new approaches to target anophelines. To maximise LLIN performance, new designs should ensure that novel chemistries or other treatments do not impair the essential attractiveness of a human-baited LLIN; indeed, efforts to enhance or exploit it should be pursued. The results might also lead to improved vector sampling, *e.g.* CDC light traps or other devices placed directly above bed nets might yield better samples^53^.

Mosquito velocities, measured here in free-flying anophelines responding to complete human hosts, were faster in the presence of the host (previously reported with wind tunnels^9^) but significantly slower (approximately 10%) when the host was protected by an LLIN. However, the velocities measured close to the bed net surface, reported here for the first time, were similar at LLINs and untreated nets. The results are significant, first, because there were no significant differences in the proportions of mosquitoes that decelerated prior to contact and the distance from the net where deceleration occurred, further evidence for the inability of *An. gambiae* to detect the LLIN, and the absence of significant repellent properties, even at close range. Secondly, they indicate that prior to contact, mosquitoes detected the presence of net barriers, including the unbaited untreated net. Landing behaviour in insects is strongly linked to visual interpretation of proximity to a surface^54^,^55^,^56^ and the eyes of nocturnally active mosquitoes like *An. gambiae* are sensitive to conditions of low visible light^57^. Tests were carried out using LEDs with a peak wavelength of 850 nm, beyond the visual perception range of *An. gambiae*^58^. Despite our efforts, we cannot guarantee that the test insectary was totally dark and it is possible that a light leak might have allowed *An. gambiae* to navigate visually. Alternatively, mosquitoes may have detected changes in air movement or the odour plume on coming in to proximity with the net surface^59^,^60^, using the Johnston’s organ or halteres, which are involved in the detection of mechanosensory cues^61^,^62^,^63^. Notably, *An. gambiae* showed similar responses by avoiding ‘invisible’ clear plastic obstacles when orienting to host cues in a wind tunnel study^64^.

Unlike coordinated landing on the tarsi, uncontrolled collision potentially could influence the quantity of insecticide deposited onto a mosquito, and we questioned whether mosquitoes were responding sufficiently far in advance to avoid ‘crashing’ into the LLIN. Deceleration started at only 0.12 seconds prior to net contact (26–41 mm from the net). We are unaware of any appropriate studies on mosquitoes for comparison, but *Drosophila melanogaster* flying at 300 mm/s began deceleration when 27 mm away from the landing point^65^, values that are remarkably similar to those measured in our study. That study also showed that the deceleration point varied with flight velocity: slower-flying *Drosophila* began deceleration closer to the landing point, also seen in our data. This provides further evidence that mosquitoes detect the presence of net barriers prior to contact.

Ongoing work will explore flight trajectories further and investigate responses in resistant malaria vector populations, other LLINs and other mosquito species. The tracking system has been deployed in rural locations in Africa where preliminary results indicate that these laboratory findings are representative of wild populations (Angarita-Jaimes *et al.* unpublished). Though still at an early stage, already these findings significantly contribute to the evidence base required for improved vector control tools by identifying previously unrecognised vector behaviours that may be vulnerable to targeting via simple interventions, and mechanisms that identify potential routes for reducing quantities of insecticide used or for the use of previously unavailable insecticide classes. They also provide a base for further research on basic behavior and much-needed studies into behavioural mechanisms of insecticide resistance. Not least, the study provides a new platform for elucidation of LLIN function and evaluation of new LLINs^66^,^67^,^68^ and other vector control tools such as spatial repellents^69^, at a rapid and cost-effective screening stage prior to larger scale testing in the field^70^.

## Methods

Three to five day old unfed adult female (25 per experiment) *An. gambiae s. str.* “Kisumu” strain, colonised at Liverpool School of Tropical Medicine (LSTM), were tested in a dedicated insectary at LSTM (5.6 m × 3.6 m in area 2.3 m high; climate controlled at 27 ± 2 °C, 70 ± 10% Relative Humidity).

The LLINs used were Permanet^®^ 2.0 (75 denier polyester net with deltamethrin at 55 mg/m^2^; Vestergaard, Lausanne, Switzerland), a WHOPES approved product^71^. Untreated nets were assembled from untreated polyester net of similar mesh.

Data were recorded and analysed from 23 laboratory tests (25 mosquitoes/test): 10 with an untreated net and 10 with an LLIN, each with 10 different human bait individuals; 3 tests used an unbaited (*i.e.* no human bait) untreated net. Ten human volunteers were used, a sample size exceeding that used in previous studies investigating similar behaviours^7^,^8^,^48^ and each volunteer was tested with an LLIN and an untreated bed net.

### Mosquito tracking

Mosquitoes were tracked using paired identical recording systems (capturing upper or lower body sections, Fig. 1B–D), each comprising a single high power infrared LED and acrylic diffuser, aligned with a pair of Fresnel lenses (mounted either side of the bed net, Fig. 1A) and monochrome camera and lens (as detailed in Supplementary Methods). The complete system captured an area of 1.2 m × 2.4 m, with blind zones of 100 mm × 1200 mm in the centre and 50 mm × 1200 mm on each side (Fig. 1B–D). Cameras were operated from a computer outside the insectary. The set-up was illuminated by two 850 nm (wavelength spectrum from 790–885 nm; invisible to humans and mosquitoes^58^) infrared light emitting diodes (1000 mA minimum; M850L2, Thorlabs, UK), one per Fresnel lens pair, located 1.2 m behind the focusing Fresnel lenses. Minimal barrel type lens distortion was observed, as assessed in multiple planes along the optical axis between the Fresnel lenses. Hence, any image distortions present would have affected the absolute positional accuracy across the entire field of view but have negligible effects on displacements when evaluated during tracking. Mosquito activity was recorded at 50 frames/second, using StreamPix software (www.norpix.com) and data saved as .seq files.

Since multiple mosquitoes were present in all tests and the entire room was not visible, determining the total number of mosquitoes responding or tracking individual mosquitoes throughout the test was not possible. Hence analyses were performed on flight tracks, and as every track theoretically could have been a different mosquito, each track from entry and exit in the field of view was analysed independently.

Segmentation and tracking algorithms were developed using bespoke software written in Matlab (Mathworks), to extract and interpret trajectory duration, time resolved velocity, distance travelled, tortuosity, and the number and duration of contacts with the bed net. Flight track segments were categorised in behavioural modes using existing quantification algorithms (Angarita-Jaimes *et al.* unpublished). A track could comprise up to three different behavioural modes (all except swooping, where no net contact occurred) and where more than one mode occurred, the times spent in each mode were recorded separately.

### Quantifying net activity

Track duration was analysed using a linear generalised linear model with normal probability distribution. Track numbers were analysed using a generalised linear model with Poisson distribution. The time lag between the first mosquito’s first appearance in the field of view (using the natural log to correct for skew) and its first contact with the net, and the effect of net type were assessed with Kaplan Meier Survival Analysis.

### Quantifying net approach

Analyses were applied only to activity recorded in the first ten minutes. The point where a track first appeared in the field of view was classed as either high (*i.e.* over the net: regions 12 and 13 Fig. 2A) or low (all other positions). Tracks starting on the net were considered likely to be fragments of incomplete tracks (*e.g.* track continuity was lost during movement between lenses or darker net regions or tracks could not be linked with confidence) and discarded; rigorously applying this rule eliminated 24% of tracks from analysis. A one-sample two-tailed *t*-test was used to compare the percentage of tracks making high approaches, with the expected value equal to 36% (the proportion of the total field of view edge, within regions 12 and 13). Location of first net contact was assessed using the definitions of contact stated in relation to activity modes *i.e.* a sharp change in track direction, or frequent semi-periodic change.

### Quantifying velocity and tortuosity

Flight velocity values were calculated using whole swooping tracks. Tortuosity values were calculated using whole swooping tracks, and track sections prior to first net contact for other flight types. To measure tortuosity, an index of the degree of flight meander, tracks were subdivided into sections comprising 40 sequential positions (average length 280 mm), and tortuosity calculated as the ratio of actual distance travelled to the straight line distance between the two end points on the section; sub-section values were then averaged to provide track value. This method removed bias resulting from extreme meandering tracks that started and ended in close proximity. Although speed and tortuosity data were not normally distributed, results from GLM analysis of transformed data were unchanged, and the untransformed data are shown.

### Determination of velocity/deceleration prior to contact

To explore mosquito velocity during approach and landing at bed net surfaces, trajectories in which mosquitoes flew for at least one second prior to contacting the net were selected, and a 65-point section of each trajectory, from 1 second before contact to 0.3 seconds after contact was selected. Instantaneous velocities and rates of acceleration/ deceleration of these segments were calculated for each track point as described in the Supplementary Methods, and the point closest to the net where deceleration started was determined. The maximum point therefore that could be classified as the start of deceleration was point 50, net contact occurred at point 51 (Fig. 3), and the track length between the deceleration start point and net contact point was calculated.

With front legs extended during flight^56^, mosquitoes potentially could have contacted the bed net with their tarsi before the tracking algorithm that detected the mosquito’s body, could detect the change of direction indicating ‘collision’. On this basis, the numbers of tracks where deceleration began within 3mm of the net surface (*i.e.* when leg contact could not be excluded) or that accelerated on their last two points of flight towards the net, were quantified for each repeat test. As our interest was in determining whether mosquitoes decelerated prior to contact, these events were classed as “contacts without deceleration” and were excluded from further analysis. Remaining tracks were used to calculate a mean track distance between the point at which deceleration occurred and the net, for each of the 23 test replicates. Average instantaneous velocity at the deceleration was calculated for each test.

### Defining and quantifying contact with a bed net surface

Bed net contacts were identified as resting tracks, or by sharp changes in mosquito flight direction at the net surface, defined as minimum angle changes of 80° in visiting mode (Fig. 1F). In bouncing mode (Fig. 1G), angle changes during repetitive contacts were often lower, and repetitive oscillations in ‘*x’* and/or ‘*y’* co-ordinates were detected from zero crossings of a bandwidth-filtered position vs. time history (Angarita-Jaimes *et al.* unpublished). To avoid spurious connections between unrelated net-arrival and net-departure tracks, resting periods were limited to a maximum of 300 seconds per event.

Time spent in contact with the net was calculated from the sum of all contacts accrued through single visits or rests, and multiple bounces. Since tracking behaviour of individual mosquitoes over the course of any test was not possible, maximum and minimum values of net contact time per mosquito were estimated as follows: the maximum value was total contact divided by the maximum number of mosquitoes observed attacking the net simultaneously in each test; the minimum value assumed that all 25 mosquitoes responded simultaneously, and calculated each mosquito’s activity as 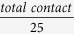. If the total number of trajectories recorded was fewer than 25 (only found in estimates for the first 10 minutes) the actual value was used as the total number of recorded trajectories.

### Localisation of activity at the bed net interface

The field of view was divided into 16 regions, ten on the net surface and six in the surrounding space (Fig. 2A). A mosquito track was assigned to regions 1–10 when contact with that region was detected. Swooping tracks in regions 1–10 were assigned to region 15 or 16 (left or right camera fields, respectively). Mosquito activity showed no bias towards either the right or left camera field (t-test, *p* = 0.523). Total activity, swooping, visiting, bouncing and resting were scaled by region area, giving values of seconds/mm^2^, to compensate for size differences between regions. Point of first net contact, and duration of net contact were analysed without scaling for area. Larger combined regions were used for analysis of point of first contact (Fig. 2B) as low numbers of data points occurred in the first ten minutes of some tests.

### Rates of mosquito activity throughout the 60 minute test period

Mosquito activity over the hour’s test was grouped in to 12 five-minute intervals. Using Prism 6, these were fitted to an exponential decay equation to find the value of the decay constant (*k*) in the equation 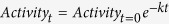 (where *t* = time in minutes).

### Statistical Analyses

Statistical analyses used SPSS Statistics 21 (IBM) and Prism 6 (GraphPad). Random effects generalized linear models with normal probability distribution were used for analyses of activity time, behavioural modes, region preferences, velocity, tortuosity, percentage of tracks contacting the net without deceleration, distance from the net and velocity of track at deceleration point, and effects of net type. Where values were not normally distributed according to calculations of skewness and kurtosis, averages were calculated as geometric means. Generalized linear models with Poisson distribution were used to assess differences in numbers of tracks and Kaplan Meier survival tests to assess differences in lag between appearance and net contact. For analyses of rates of activity decay over time, *k*-values were tested for significant differences between untreated nets and LLINs using GLM and 95% confidence intervals used to determine time intervals with significant differences in activity (Fig. 4A). For all tests, the α threshold used was 0.05. In all cases except for ‘all treatment’ data 95% confidence intervals were calculated using the *t* distribution to account for small replicate numbers. Unless stated otherwise, data are reported as arithmetic means and 95% confidence intervals.

### Ethical Permission

Methods were carried out in accordance with the approved guidelines. Informed consent was obtained from all participating human subjects. The study was approved by Liverpool School of Tropical Medicine Research Ethics Committee (‘Behaviour of African malaria vectors’: Permit no. 12.13, issued 24^th^ May 2012).

## Additional Information

**How to cite this article**: Parker, J.E.A. *et al.* Infrared video tracking of *Anopheles gambiae* at insecticide-treated bed nets reveals rapid decisive impact after brief localised net contact. *Sci. Rep.* **5**, 13392; doi: 10.1038/srep13392 (2015).

## Supplementary Material

Supplementary video

Supplementary Information

## Figures and Tables

**Table 1:** Total activity time of *Anopheles gambiae* recorded in each behaviour mode.

	N	Swooping	Visiting	Bouncing	Resting
Unbaited	3	7.5 (0.5–116.1)	10.6 (1.2–96.2)	0.5 (0–22.1)	0.1 (0–20.0)
		ab	a	a	a
Untreated	10	7.7 (6.1–9.8)	33.2 (24.0–46.1)	70.1 (57.7–85.1)	10.3 (7.0–15.3)
		a	b	b	b
LLIN	10	3.4 (1.9–6.2)	6.9 (3.5–13.6)	7.7 (3.1–18.7)	2.0 (0.8–5.0)
		b	a	c	c

Total duration of all tracks classed in each behaviour mode over 60 minute tests (geometric mean and 95% confidence interval, minutes). Since multiple mosquitoes were often active simultaneously in the field of view, the total activity times could exceed 60 minutes. Values for each mode followed by the same letter are not significantly different at *p* < 0.05 (Generalized Linear Models), between different net types.

**Table 2:** Duration of *Anopheles gambiae* contact with bed nets.

Duration of physical contact with the bed net surface (60 min test)				
	N	Mean total time (all contacts)^1^ (min)	Mean time/mosquito (25 mosquitoes)^2^ (s)	Mean time/mosquito (observed max number)^3^ (s)
Unbaited	3	2.4 (−2.1–6.8)^a^	5.7 (−5.1–16.4)^a^	41.9 (−19.5–103.4)^a^
Untreated	10	33.1 (24.3–41.2)^b^	79.5 (58.2–100.7)^b^	334.1 (264.6–403.6)^b^
Treated	10	7.3 (3.9–10.7)^c^	17.5 (9.3–25.7)^c^	95.6 (57.9–133.2)^c^

Mean duration of contact with the bed net surface for 60 minutes tests, as calculated for

^1^mean total of all contacts observed.

^2^mean contact time per mosquito assuming all 25 mosquitoes responded.

^3^mean contact time per mosquito based on the maximum number of individual mosquitoes observed simultaneously (mean and 95% confidence interval), values for each mean followed by the same letter are not significantly different between net types at *p* < 0.05).
