# Supplementary material for: Infrared video tracking of Anopheles gambiae at insecticide-treated bed nets reveals rapid decisive impact after brief localised net contact
Source: Sci Rep. 2015 Sep 1;5:13392. doi: 10.1038/srep13392 (PMC4642575; doi:10.1038/srep13392)
Supplement: Supplementary Information [file srep13392-s2.pdf]

Infrared video tracking of *Anopheles gambiae* at insecticide-treated bed nets reveals rapid decisive impact after brief localised net contact

Josephine E.A. Parker, Natalia Angarita-Jaimes, Mayumi Abe, Catherine E. Towers, David Towers & Philip J. McCall

SUPPLEMENTARY METHODS

Mosquitoes

Mated unfed adult female *An. gambiae s. str.* Kisumu strain were obtained from a long-established colony at the Liverpool School of Tropical Medicine (LSTM) maintained on human blood, under a L12:D12 hour light: dark cycle. To avoid potential impact of age on responses to insecticide, all insects used were three to five days post-eclosion. Mosquitoes were starved of sugar and water for 4-6 hours, and introduced into the experimental room at least 1 hour before testing. All tests started after the first hour of the scotophase.

Insectary environment

The study was carried out in a dedicated insectary at LSTM, measuring 5.6m x 3.6m in area and 2.3m high, with a controlled climate (27±2˚C, 70±10% Relative Humidity). One hour before tests, the volunteer entered the bed net, the mosquitoes were placed in a paper cup connected to an external release cord, suspended against the wall at a height of 2m (chosen to simulate entry at eave height), and the room was closed. The mosquito release point was located 1.4m from the end of the net.

To avoid any influence of air movements or climate gradients, humidification and air conditioning were switched off. At the end of the 60-minute test period, mosquitoes in the room were collected with aspirators. Between tests with treated and untreated nets, surfaces in the insectary were washed (5% Decon 90) and rinsed and air vented with a fan in the doorway.

Long-lasting insecticidal bed nets (LLIN)

LLINs were removed from packaging and hung (in a separate room) for four weeks prior to tests. To facilitate image capture, bed nets were altered and sewn to fit the mattress tautly to eliminate wrinkling or folding, and the top surface of the net was tilted on its long axis (measuring 750mm and 450mm high on opposite sides; Figure 1A). Human volunteers lay on a fresh sheet on a 2m x 0.88m mattress (180mm thick, 480mm above the floor at the top) on timber slats mounted on bricks to ensure rigidity and reduce vibration.

Human baits and test conditions

Volunteers (5 males and 5 females of different ages and a range of ethnicities) were recruited from staff and students at the Liverpool School of Tropical Medicine. Each volunteer was tested once with Permanet 2.0 and once with the untreated net. Volunteers were clothed but barefoot and lay on their backs, as immobile as comfort permitted, to avoid influencing mosquito movement or generation of spurious tracks by the motion detection software. All were asked to refrain from using scented toiletries on the morning prior to testing. To control for any influence of body orientation, half the participants were randomly assigned to one position (*i.e.* 50% with head and 50% with feet towards the mosquito release point), which they retained for both tests. Volunteers were tested with LLIN or untreated net on different days, with an average interval of 13 days between tests.

Mosquito tracking system

Each of the paired identical recording systems comprised a 12.5mm imaging lens (Kowa LM12HC 1"; Multipix UK) mounted on a monochrome camera (Baumer HXC40NIR, Camera Link, 4Mpix; Lambda Photometrics, UK), a pair of Fresnel lenses (1400 x 1050mm and 3 mm thick; NTKJ Co., Japan) and a single high power infrared LED (850nm, 1000mA minimum; M850L2, Thorlabs, UK) placed behind a 3mm thick acrylic diffuser (Comar Optics, UK). Components were mounted on heavy tripods or aluminium frames to minimise sagging and movement. Fresnel lenses had a focal length of 1.2m and were positioned 1.75m apart to

accommodate the width of the bed and mattress, with a gap of approximately 43cm between the lens and mattress on each side (Figure 1A).

In this back-lit set-up, the large aperture Fresnel lens enabled the illumination source to be formed into a large area approximately collimated beam. This allowed optically efficient illumination of the large volume from a single light source (the infrared LED). Moreover, an additional diffuser was placed between the light source and the Fresnel lens to help homogenise the illumination across the entire field of view. This also ensured that the LED source was not directly imaged. The efficiency of the illumination enabled the exposure time of each frame to be reduced to typically 3 milliseconds, thus ensuring that the image was not overexposed.

Both cameras were operated by a single computer (Intel Core i7. 3.4 Ghz.8 Gigabytes RAM, Windows 7 Ultimate; 10 hard drives at 2 Terabytes each, 5 drives per camera).

Determination of *An. gambiae* velocity and rate of deceleration prior to net contact

To analyse tracks contacting the net a sub-set of trajectories was selected in which mosquitoes flew for at least one second, and then made contact with the net. A 65-point section of the trajectory, starting 1 second prior to contact and extending 0.3 seconds after contact was selected for further analysis. Velocity of these tracks was calculated at each of the points along its length using the equation $v_i = \frac{\|\overrightarrow{r_{i+1}} - \overrightarrow{r_{i-1}}\|}{t_{i+1} - t_{i-1}}$ where v_i is the velocity at point *i*, r_i is the position vector at point *i*, and t_i is time stamp at point *i*. Velocities for each track were filtered with a low pass 3rd order Butterworth filter, with a cut-off frequency of 11.25Hz and a sampling frequency of 50Hz.

Acceleration at each point was calculated using $a_i = \frac{v_{i+1} - v_{i-1}}{t_{i+1} - t_{i-1}}$ where a_i is acceleration at point *i*. The track point at which pre-contact deceleration started was calculated using methods modified from [51]. An algorithm starting at the point of collision worked backwards along the track to identify the first incidence of two consecutive points with positive acceleration values.

The point following this (i.e. closer to the net) was used as the start of deceleration in the track. Using this system the maximum point that could be classified as the start of deceleration was point 50 (contact occurs at point 51). The remaining track length between deceleration point and contact point was determined for each track.

Supplementary Video: Mosquito flight at a human-occupied bed net in swooping, visiting, bouncing and resting behavioural modes

The video demonstrates the characteristic movement patterns within the different behavioural modes: in swooping, mosquitoes fly without contacting the net; visiting flights make infrequent net contacts; bouncing mosquitoes make frequent short persistent attacks on the net surface. In resting the mosquito is stationary, or slow moving. During the resting video, the marker disappears when movement ceases (start of resting) and reappears at the same point when movement restarts (resting mode ends). As markers are attached to moving objects, the mosquito is not highlighted when it stops moving, though the tracking algorithm continues to follow its position.
